# From Pathways to Patients in Atopic Dermatitis: Advanced Systemic Therapies

**DOI:** 10.3390/ijms262311487

**Published:** 2025-11-27

**Authors:** Alvaro Prados-Carmona, Husein Husein-ElAhmed, Francisco José Navarro-Triviño, Ricardo Ruiz-Villaverde

**Affiliations:** 1 Department of Dermatology, Hospital Universitario San Cecilio, 18016 Granada, Spain; ismenios@hotmail.com; 2 Instituto Biosanitario de Granada, ibs.Granada, 18012 Granada, Spain; 3 Escuela Internacional de Posgrado, Universidad de Granada, 18012 Granada, Spain; 4 Department of Dermatology, Hospital de Baza, 18800 Granada, Spain; huseinelahmed@hotmail.com; 5 Unit of Contact Eczema and Immunoallergic Diseases, Hospital Universitario San Cecilio, 18016 Granada, Spain; fntmed@gmail.com; 6 Department of Medicine, University of Granada, 18016 Granada, Spain

**Keywords:** atopic dermatitis, pathophysiology, immunology, systemic treatment, review

## Abstract

Atopic dermatitis is the most prevalent chronic inflammatory skin disease, posing a significant individual and healthcare burden. Traditionally managed with topical agents and broad immunosuppressants, the treatment landscape has shifted significantly in recent years. This review explores the transition from immunopathogenic understanding to personalized treatment strategies through advanced systemic therapies. We provide a thorough description of the current therapeutic arsenal, including approved monoclonal antibodies and Janus kinase (JAK) inhibitors, as well as experimental agents under clinical investigation. Key cytokines and receptors implicated in type 2 inflammation are explored alongside relevant intracellular signaling pathways. Special attention has been given to literature published from 2015 onwards. By synthesizing the latest scientific and clinical knowledge, this review aims to provide clinicians with practical guidance for navigating the evolving landscape of atopic dermatitis management and improving patient outcomes.

## 1. Introduction

Historically, apart from the use of emollients, therapeutic approaches for atopic dermatitis (AD) primarily focused on alleviating its symptoms or preventing lesions through corticosteroids or broad immunosuppression. However, recent advances have shifted the focus towards immunomodulating the specific causes of the disease [1,2,3,4]. This shift marks a significant turning point in the management of AD, switching the focus from the manifestations of the disease to its underlying mechanisms [5]. This correlates with the many novelties that are revolutionizing the dermatology landscape of immune-mediated diseases [6].

The relevance of providing more tailored, effective treatments to our patients is unchallenged, especially considering that AD presents a significant burden, not only to our patients’ quality of life but also to healthcare systems [7]. Despite the higher initial cost of some of the newer alternatives, these therapies are expected to yield long-term economic benefits by reducing direct medical costs associated with disease progression, therapeutic adverse effects, and the need for complex follow-up or regular monitoring as well as indirect costs, such as lost productivity and diminished quality of life [8,9].

## 2. Materials and Methods

**Figure 1:**
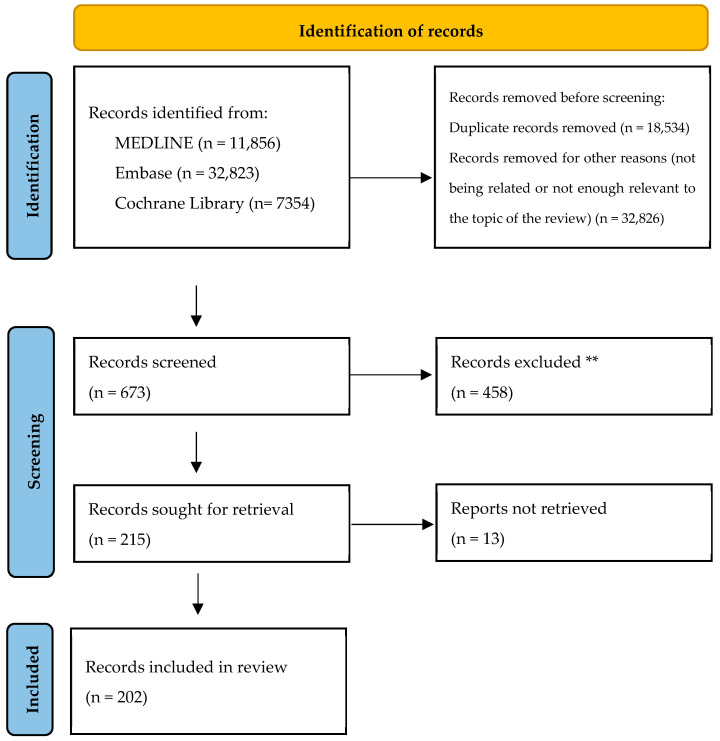
PRISMA flow diagram of entries assessed. ** No automation tools were used to exclude records.

Relevant English- and Spanish-language articles, with special focus on those from 2015 onwards, were collected between November 2024 and April 2025 for this narrative review. A comprehensive search was conducted using the electronic databases MEDLINE (via PubMed), Embase (via Elsevier: embase.com), and the search engine Cochrane Library, complemented by manual screening of reference lists from retrieved papers. Keywords used included “atopic dermatitis” and “physiopathology” or “treatment”, “immunological targets”, “interleukin”, “new therapies”, “systemic advanced therapies”, “real-world”, “clinical trial”, “biologic therapy”, “JAK inhibitor”, “OX40/OX40L”, “pipeline”, and related variations. Results were filtered by article type and relevance to the topic. Additional references were retrieved from them (Figure 1).

Some figures were created by the authors using BioRender (Science Suite Inc., Toronto, ON, Canada, available at https://app.biorender.com, accessed on 16 November 2025) with permission to publish. During the manuscript preparation process, the authors used ChatGPT-4o (OpenAI, San Francisco, CA, USA) under human oversight to improve the clarity and readability of certain sections. The final content was reviewed, edited, and fully approved by the authors, who take complete responsibility for its integrity.

## 3. Therapeutic Landscape: Overview of Main Targets, Approved and Experimental Molecules

**Figure 2:**
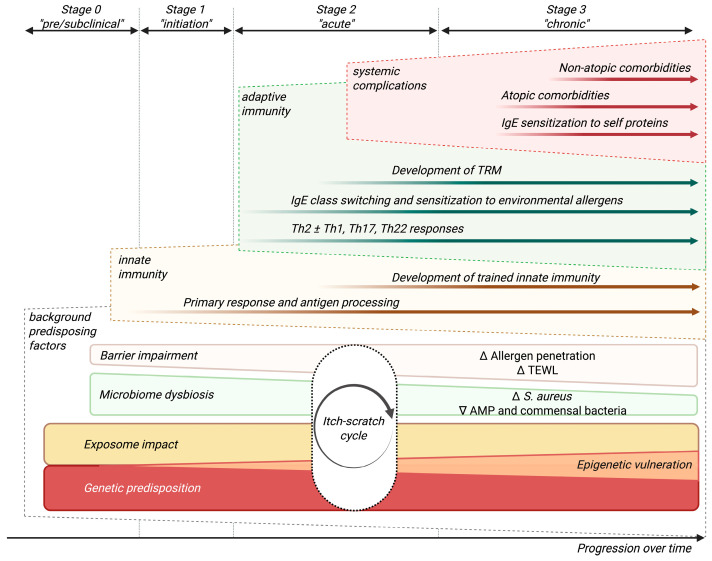
Multi-dimensional model of AD as a high-level overview of disease etiopathogenic factors and natural progression. The figure virtually outlines four different stages of progression over time, rather than of individual lesions: Stage 0 (pre/subclinical), Stage 1 (initiation), Stage 2 (acute), and Stage 3 (chronic). Disease progression begins with the interaction between genetic predisposition and exposome impacts, which further drive the accumulation of epigenetic vulnerabilities. These background risk factors lay the foundation for barrier impairment, microbiome dysbiosis (*Staphylococcus aureus* overgrowth, reduced levels of antimicrobial peptides [AMPs] and commensal bacteria) and the establishment of the itch–scratch cycle. In Stage 1, innate immunity is the primary driver, with early antigen penetration and immune response elicitation in the context of skin barrier disfunction and release of alarmins. By Stage 2, AD becomes clinically evident. Adaptive immunity becomes dominant, marked by Th2 activation, immunoglobulin IgE class switching, and sensitization to environmental allergens, alongside contributions from other Th subsets depending on the disease phenotype. In Stage 3, further complications arise, including IgE sensitization to self-proteins, atopic comorbidities (e.g., asthma, allergic rhinitis…), and non-atopic comorbidities. The figure also emphasizes the roles of trained innate immunity and tissue-resident memory cells (TRM), which have gained attention in recent years. This framework connects immune dysregulation, environmental factors, and skin barrier dysfunction to the clinical trajectory of AD.

AD results from a combination of genetic, environmental, immunologic, and barrier function-related factors [5,10]. Despite the complex conceptualization of this entity, for didactic purposes, we have elaborated a simplified multidimensional model of its pathogenesis that schematically covers the various factors involved over the course of the disease (Figure 2).

**Figure 3:**
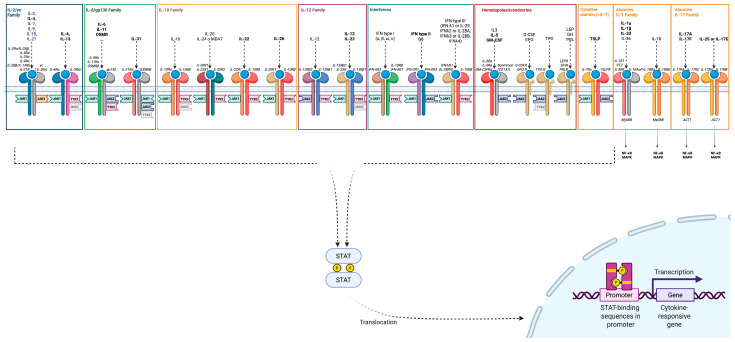
Common immune mediators and their mechanism of action. Different cytokines, belonging to different families, can contribute to disease development in AD (some that are more relevant have been written in bold letters to facilitate their identification). Most activated receptors signal via the Janus kinase (JAK)/STAT cascade, each with concrete JAK subtypes (JAK1, JAK2, JAK3 and TYK2). If a JAK protein is depicted with a faint color next to a receptor, this indicates that its activity within the pathway has been reported only in murine models, experimental conditions, non-physiological contexts, or specific cellular subsets. The signal transducer and activator of transcription (STAT) family consists of seven members. The JAK/STAT pathway is used by many cytokines relevant to AD apart from interleukins (ILs) themselves, including alarmins and interferons (IFN). Sharing intracellular pathways makes some functions redundant and explains the complexity in managing AD effectively by addressing a unique molecule. For example, the fact that the IL-7Rα is a shared component between the thymic stromal lymphopoietin (TSLP) receptor complex and the IL-7 receptor complex explains some of the overlapping functions of these cytokines. However, whereas the TSLP receptor complex utilizes JAK1 and JAK2 for STAT5 activation, IL-7 signaling involves JAK1 and JAK3 [11]. Highlights from innate immunity: TSLP interacts with its receptor complex (TSLP receptor [TSLPR]/IL-7 receptor alpha chain [IL-7Rα]) on various immune cells and is particularly crucial in the pathogenesis of AD. For instance, it induces the expression of OX40L on dendritic cells (DCs), a key signaler for the polarization of naïve T cells towards a T helper (Th) 2 phenotype. TSLP also elicits innate lymphoid cells (ILC) 2, together with but also independently from IL-33 [12]. IL-33 belongs to the IL-1 superfamily—which also comprises the IL-1, IL-36 and IL-18 subfamilies [13]—and is mainly secreted by structural cells, and binds to a specific receptor from the Toll-like receptor (TLR)/IL-1R superfamily. It acts as an amplifier towards ILC2 and Th2 cells, stimulates the production of IL-4 by eosinophils, increases local fibrosis, and has replicated AD-like cutaneous manifestations with eosinophil and mast cell accumulation in murine models [14,15,16,17]. Notably, both TSLP and IL-33 influence mast cell proliferation (IL-33 acting via the MyD88 pathway and TSLP involving STAT6 signaling), adhesion, and mediator release [18]. IL-25 similarly promotes Th2 differentiation through DCs and ILCs [19]. Highlights from adaptive immunity: Cytokines such as IL-1α and IL-36 help facilitate transition to adaptive immunity. IL-4 is essential for Th2 differentiation. IL-4 and IL-13 receptors will be addressed more deeply in a separate figure due to their central role in disease pathogenesis and the development of various targeted therapies against them or their receptors. IL-5 is crucial for eosinophil maturation and survival. IL-31, despite signaling through its own receptor, belongs to the IL-6 family, and is related to inflammatory responses and pruritus. IL-22 has a role in barrier repair but can also contribute to skin lichenification in chronic stages of the disease. IL-2 and IL-10 signaling contribute to downregulation of immune responses through regulatory T cells (Tregs) and are decreased in AD. IFN-γ is produced by Th1 cells and increases its relevancy in chronic stages of the disease with a higher Th1 presence.

Above all, AD is defined by the dysregulation of the immune system. Many mediators, belonging to different families, are progressively better characterized as we understand more of the immunology underlying not only AD, but many common inflammatory dermatoses (Figure 3).

Treatment of AD begins with patient education about AD’s chronic, relapsing nature and involves skin barrier care, topical corticosteroids for flares, and proactive maintenance with topical corticosteroids or calcineurin inhibitors for chronic cases [20]. A Delphi consensus between dermatologists and hospital pharmacist insisted on improving patient education on treatment adherence and non-pharmacological measures, while restricting systemic immunosuppressants to severe or refractory cases [21]. Many treatments—topical and systemic, older and newer—have proven clinically and molecularly to be beneficial for AD [22,23,24,25]. However, the therapeutic landscape of AD has evolved significantly with the identification of specific immunological targets [26]. As such, we are shifting from treating the consequences and clinical manifestations of AD to addressing its concrete causes, and potentially to disease course modification. Also, the development of targeted therapies is especially appealing as the widespread adoption of these drugs following inclusion in clinical guidelines, together with eventual patent expirations and the development of biosimilars—as in psoriasis [27]—will likely drive increased affordability over time.

**Figure 4:**
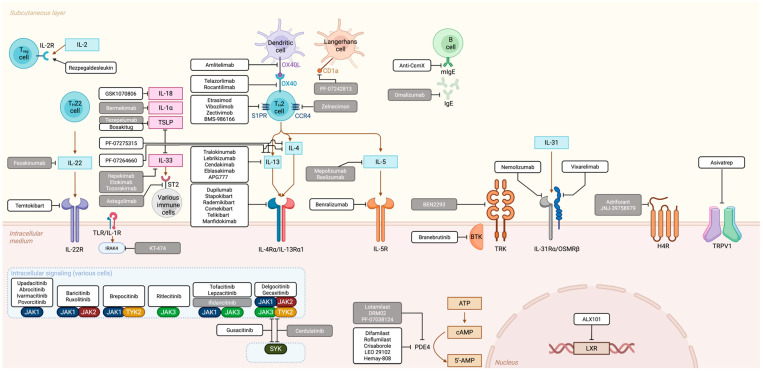
Key immunological targets and overview of advanced therapeutic therapies designed for modulating these actors either in AD or other inflammatory diseases. Gray alternatives are those that have allegedly been discontinued, whereas in white are ones are either already available or still under ongoing development. Cytokines in pink boxes are alarmins.

Currently, available molecules in Europe include: dupilumab, tralokinumab, lebrikizumab, abrocitinib, baricitinib, and upadacitinib. Additionally, nemolizumab has recently been also approved for both prurigo nodularis and AD. They will be covered in the following subsections according to their mechanism of action, alongside other relevant investigational drugs that have been or are being studied (Figure 4).

### 3.1. “T-Cell Inhibitor” Family

AD is defined by immune dysregulation, both systemically and on lesional skin. T helper (Th) 2, together with Th22 activation, with additional contributions from Th17 and Th1 pathways, are definitory of the disease, showcasing a heterogeneity that goes far beyond other well-studied dermatoses like psoriasis [28]. Several medications have been designed to target the cytokine pathways driving T cell dysregulation, which is key for disease development.

The adaptive immune response in AD is initially dominated by a Th2 skew, particularly evident in the acute phases of the disease (Figure 1) [5,28]. As an overview, interleukin (IL)-4 impacts skin barrier function by downregulating the expression of key structural proteins in keratinocytes such as filaggrin, plus it initiates and sustains the Th2 response, recruiting immune cells like eosinophils and mast cells and promoting IgE class switching in B cells; IL-13 maintains Th2 inflammation and drives fibrosis and skin remodeling; IL-31 is a primary mediator of pruritus, bridging immune and neural pathways [29,30,31,32]; IL-5 activates eosinophils; and IgE antibodies perpetuate sensitization together with mast cells, basophils, and other innate immunity agents, triggering allergic inflammation pathways upon subsequent allergen exposures. All of them contribute to the spectrum of defining characteristics of the disease, including barrier defects, *S. aureus* colonization, and systemic inflammation [5,26].

**Figure 5:**
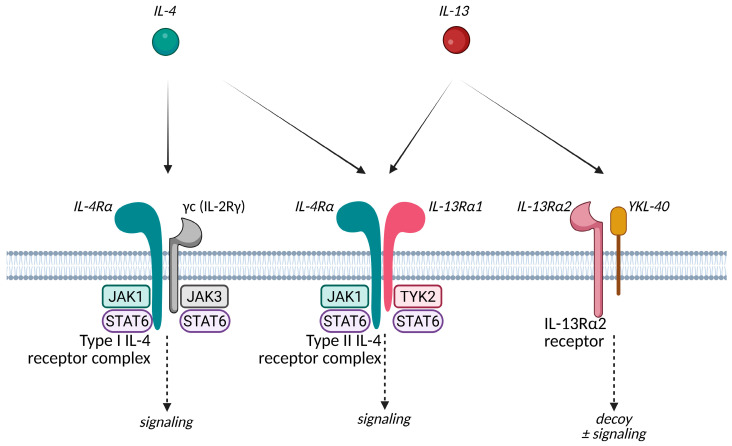
IL-4 and IL-13 membrane receptor complexes and intracellular signaling pathways. IL-4 signals through two receptor complexes: the Type I IL-4 receptor complex, comprising IL-4Rα and the common gamma chain (γc or IL-2Rγ), and the Type II IL-4 receptor complex, comprising IL-4Rα and IL-13Rα1. IL-13 primarily signals through the Type II receptor complex but can also interact with IL-13Rα2, which serves as a decoy receptor with limited downstream potential [40]. Type I IL-4 receptor complex is primarily expressed on hematopoietic cells [33,34]. Additionally, this receptor is also expressed on human conjunctival epithelial cells and corneal fibroblasts, where it induces the production of mucins by globet cells and other immune-modulatory functions, contributing to the pathophysiology of allergic conjunctivitis and other ocular surface disorders [41,42]. Therefore, the type II IL-4 receptor complex is the primary receptor involved in the pathogenesis of AD, contributing to barrier dysfunction and the recruitment of Th2 cells due to its widespread expression on non-hematopoietic cells, including keratinocytes, fibroblasts, and immune cells [34,43,44]. Activation of the type II IL-4 receptor complex leads to the phosphorylation of Janus kinases (JAK1, bound to the IL-4Rα chain, and TYK2, associated with the IL-13Rα1 chain) and subsequent activation of signal transducer and activator of transcription (STAT) 6, which regulate the expression of genes driving type 2 inflammation. IL-13Rα2 exists in both membrane-bound and soluble forms, acting as a high-affinity decoy receptor for IL-13. Unlike the IL-4Rα/IL-13Rα1 receptor complex that transmits pro-inflammatory signals, IL-13Rα2 binds IL-13 with high affinity but does not activate downstream inflammatory pathways, thereby modulating the excessive immune responses characteristic of AD. Silencing IL-13Rα2 in the HaCaT keratinocyte cell line significantly amplifies IL-13-mediated STAT6 activation compared to cells treated with control small interfering RNA, further supporting the role of IL-13Rα2 as a decoy receptor for IL-13 [45]. In human keratinocytes, IL-13Rα2 expression is upregulated by IL-13, IL-4, and TNF-α, but not by IFN-γ (which does upregulate this receptor in bronchial epithelial cells), whereas IL-13Rα1 expression remains stable and unaffected by these cytokines [44,45,46,47]. Therefore, its expression is significantly upregulated in the lesional skin and serum of patients with AD and is permanently induced by mechanical scratching and pro-inflammatory cytokines as a compensatory mechanism against the overwhelming effects of IL-13, sequestering IL-13 and inhibiting further action [44,45]. Apart from withdrawing IL-13 without transmitting pro-inflammatory signals, it is also believed to attenuate fibrosis through reducing collagen and TGF-β production [44]. Studies have shown that IL-13 upregulated collagen and TGF-β1 production in fibroblasts but not in IL-13 Rα2-adenovirally transfected fibroblasts [48]. Despite being intended to reduce TGF-β, the same IL-13Rα2 and IL-13 have also been described to increase TGF-β1 through pathways involving the TMEM219 molecule, a similar mechanism used by other ligands such as chitinase 3-like 1 (CHI3L1) (also known as human homolog YKL-40) [49,50]. This still must be further elucidated but it is becoming clear that this receptor has various roles beyond its decoy function. What is clear is that despite mechanical scratching and inflammation enhancing IL-13Rα2 expression and mitigating some IL-13-mediated effects, it is not enough to fully prevent local disease.

IL-13 and IL-4 share some overlapping biological functions due to their ability to signal through this specific type II IL-4 receptor complex, which is composed of IL-4Rα and IL-13Rα1 [33,34,35] (Figure 5). IL-4 signaling cascade results in downregulation of key epidermal barrier proteins, modulation of epithelial cells and fibroblasts, enhancing IgE class switching in B cells, stimulation of dendritic cells (DCs), and upregulating the expression of chemokines (CCL17, CCL22, and CCL26) which recruit CCR4+ Th2 cells, eosinophils, and other inflammatory cells to the skin, perpetuating the inflammatory response [35,36]. It also increases the expression of other pro-inflammatory cytokines like IL-5, IL-13, IL-19, IL-20, and IL-25, and downregulates the expression of AMPs such as S100 proteins and defensins. Additionally, IL-4 decreases the number and function of regulatory T cells (Tregs) by reducing cytotoxic T-lymphocyte-associated protein 4 (CTLA-4 or CD152) expression, impairing their ability to suppress Th2-mediated inflammation [37]. It also directly enhances Th2 responses by acting on innate lymphoid cells (ILC) 2 and skin-derived DCs, promoting their capacity to polarize T cells into Th2 cells, driving both local and systemic allergic responses [38]. Mice lacking IL-4 or IL-4 receptor signaling in DCs exhibit impaired Th2 responses and reduced allergic skin inflammation [39]. Mechanical skin injury, such as scratching, induces basophil-dependent upregulation of IL-4 expression in human and mouse skin.

On the other hand, recent large-scale transcriptomic analyses highlight the dominant role of IL-13 in AD lesional skin, where IL-4 expression is nearly absent [51]. IL-13 is predominantly produced in the skin by tissue-resident ILC2s and upregulated after scratching—it can also be produced by mast cells in response to keratinocyte-derived IL-33 after mechanical skin injury [52]—and in more severely affected skin. Structurally, IL-13 downregulates the OVOL1–FLG axis, impairing the expression of FLG, LOR, and IVL, contributing to barrier malfunction and enhancing itching sensations [40,53,54,55,56]. IL-13 also upregulates periostin, a matricellular protein that further stimulates keratinocytes to produce IL-24, contributing further to FLG downregulation and barrier dysfunction [54]. It is also considered a profibrotic cytokine responsible for AD-related fibrosis [44,57]. When IL-13 and IL-4 are neutralized independently, it seems that IL-13 is the dominant cytokine in driving fibrosis [58]. Regarding its immunomodulatory functions, we should highlight its impact on mast cell activity and, together with IL-4, the stimulation of DCs and fibroblasts to produce CCL17 and CCL22, which recruit CCR4þ Th2 cells into the local tissue [38,59]. IL-13 also suppresses IL-12 production in skin-derived DCs by downregulating the expression of the IL-12p40 subunit. This suppression reduces the ability of DCs to promote IFN-γ production by CD4+ T cells, thus inhibiting Th1 cell polarization. Minimizing Th1 responses contributes to the disbalance of immune responses toward a Th2-dominated immunity. This correlates with the theory that localized blockade of IL-13 may help restore the Th1/Th2 balance in AD patients [52].

Eosinophilic allergic inflammation, which is a hallmark of Type 2 immune responses, is largely dependent on IL-5. IL-5 is produced primarily by Th2, ILC2, and mast cells. IL-5 is a crucial cytokine for differentiation, maturation, and survival of eosinophils in the bone marrow and their subsequent recruitment to inflamed skin via upregulated eotaxins (CC chemokine subfamily of eosinophil chemotactic proteins) such as CCL11 and CCL24 [60]. IL-5 signals through the IL-5 receptor, which activates JAK2, leading to the phosphorylation and activation of STAT5 (Figure 2). STAT5 then moves to the nucleus to drive the expression of genes that promote eosinophil survival and function. Additionally, IL-5 can engage the phosphoinositide 3-kinase (PI3K)/AKT pathway, which further supports eosinophil survival by inhibiting apoptosis and enhancing migration. The combined activation of these pathways by IL-5 results in sustained eosinophil-mediated inflammation, contributing to tissue damage and the chronic systemic inflammatory state in AD.

Another interesting Th2 primarily derived interleukin is IL-31. It is closely associated with other Th2 cytokines such as IL-4 and IL-13. IL-31 is increased in serum in AD and its levels correlate with disease severity [61,62]. Its receptor complex includes IL-31 receptor alpha (IL-31Rα) and oncostatin M receptor beta (OSMRβ). It induces the activation of eosinophils and keratinocytes and promotes the production of chemokines such as CCL17 and CCL22, which once again recruits Th2 cells to the site of inflammation [29,32].

#### 3.1.1. IL-4 and IL-13 Related Agents

Dupilumab, tralokinumab, and lebrikizumab block cytokines critical to Th2-driven inflammation. They are well-tolerated and effective across different AD populations, but especially in classical Th2-dominant backgrounds [63]. As such, they—and particularly dupilumab—are allegedly preferred for patients with allergic comorbidities and a presumed Th2 dominant immunotype (e.g., pediatric AD), especially if there are risk factors for thromboembolism or malignancy [63].

Dual IL-4/IL-13 inhibitor, dupilumab, was the first biologic drug used in this disease [25]. It targets the shared component for IL-4 and IL-13 receptors IL-4Rα blocking both molecules, whereas IL-13 inhibitors such as tralokinumab and lebrikizumab selectively bind to IL-13. Both approaches have been proven effective and there is still a lot of research focused on these targets. For example, the dual nature of IL-13Rα2—as a regulator of inflammation and local fibrosis, but also as a potential contributor to fibrotic remodeling when interacting with alternative ligands—presents intriguing opportunities for further innovation [44].

The dupilumab approval process was significantly supported by two phase 3 trials, SOLO1 and SOLO2, which demonstrated its efficacy and safety in adults with moderate-to-severe AD inadequately controlled by topical treatments [25]. In these trials, patients receiving dupilumab showed significant improvements in the IGA and EASI scores compared to placebos. Specifically, 37–38% of patients treated with dupilumab achieved clear or almost clear skin (IGA score of 0 or 1) compared to 8–10% in the placebo group [25,64]. Previously, an earlier phase 2b trial also provided evidence of dose-dependent improvements in clinical indices, biomarker levels, and the transcriptome of patients with moderate-to-severe AD. So far, we have already accumulated a fair amount of experience with dupilumab in various scenarios, including real world data (RWD) from different sources and use cases proving sustained efficacy and safety for long-term management of moderate-to-severe AD [65,66,67,68,69,70,71,72,73,74,75,76,77,78]. Dupilumab, despite reducing IL-4/IL-13 signaling through type I and II IL-4 receptor complexes, also has managed to normalize IL-13Rα2 levels in AD patients [22,77,79]. Since dupilumab inhibits both IL-13 signaling and improves pruritus, it is suggested that the therapy may suppress IL-13Rα2 expression driven by both IL-13 and scratching [25,44,77].

Stapokibart (CM-310) is another humanized monoclonal antibody targeting the IL-4Rα that has concluded its development and has launched in China. In a pivotal phase 3 trial, 66.9% of patients receiving stapokibart achieved ≥75% improvement in EASI-75 at week 16, compared to 25.8% in the placebo group. Additionally, 44.2% of patients achieved an IGA score of 0/1 with a ≥2-point reduction, compared to 16.1% in the placebo group [80]. Long-term data also shows sustained efficacy at 52 weeks, with EASI-50/75/90 response rates of 96.3%, 87.9%, and 71.0%, respectively [81]. Common treatment-emergent adverse events included upper respiratory tract infections, conjunctivitis, and injection site reactions, similar to those of dupilumab [80,81,82]. It has not received regulatory approval for clinical use in Europe yet. Manfidokimab (AK-120), rademikibart (CBP 201) [83], comekibart (MG-K10) and telikibart (GR-1802) are similar molecules also under investigation, with ongoing phase-III clinical trials or already in preregistration in some countries.

Tralokinumab is a human IgG4 monoclonal antibody that neutralizes free IL-13, reducing its signaling through IL-13Rα1 within the type II IL-4 receptor complex, and subsequent downstream activation of the STAT6 pathway implicated in keratinocyte dysfunction and barrier impairment. Unlike IL-4/IL-13 dual inhibitors, such as dupilumab, tralokinumab selectively targets IL-13, offering a more focused approach to type 2 cytokine blockade. Its approval for the treatment of moderate-to-severe AD in adults was supported by robust evidence from pivotal phase 3 trials, including ECZTRA 1 and ECZTRA 2. RWD on tralokinumab is emerging and supports its sustained efficacy and safety in the long-term. Recent observational studies and extension trials have confirmed improvements in disease control and quality of life, with continued reductions in pruritus. The affinity of the IL-13Rα2 decoy receptor towards free IL-13 is higher than the affinity of tralokinumab for the same IL-13. IL-13Rα2 binds IL-13 with an exceptionally high affinity, in the femtomolar range, as demonstrated by Lupardus et al., who reported a binding affinity of approximately 20 pM [84]. This high affinity is due to the larger and more complementary interface between IL-13 and IL-13Rα2. In contrast, tralokinumab, binds IL-13 with high affinity but not as high as IL-13Rα2. Therefore, the binding of tralokinumab to IL-13 effectively prevents IL-13 from interacting with both IL-13Rα1 and, partially, IL-13Rα2 [85].

Lebrikizumab is another IgG4 antibody that also exerts its effects by binding with high affinity and a slow off-rate to make IL-13 soluble in an epitope that overlaps with the IL-4Rα binding site, avoiding signaling through the IL-4Rα/IL-13Rα1 heterodimeric receptor. It does not affect IL-13 pairing with IL-13Rα2, even partially, facilitating its internalization and clearance [86]. Its approval for moderate-to-severe AD in adults was based on the results of pivotal phase 3 trials including ADvocate 1 and ADvocate 2, which demonstrated significant efficacy and a favorable safety profile. Ongoing head-to-head comparisons and real-world studies will better delineate its specific niche within the therapeutic landscape.

Experimental molecules targeting IL-13 include anrukinzumab (IMA-638; PF-05230917), cendakimab (ABT-308; CC 93538), eblasakimab (ASLAN 004; CSL 334; MK 6105), and APG777 (PR-004). Anrukinzumab was studied for asthma and discontinued from the pipeline of Pfizer. Cendakimab allegedly operates by targeting free IL-13 in a similar way to tralokinumab, whereas eblasakimab targets IL-13Rα1 and could block both IL-13 and IL-4 signaling through type 2 receptor complexes [87,88]. Both have completed phase-II development trials, and eblasakimab is moving towards phase-III. APG777 is currently in phase II of development (NCT06395948) and has an extended half-life of 77 days according to data from the phase I trial.

All approved drugs targeting type 2 cytokines account for a favorable safety profile, making them suitable for long-term use [89,90,91,92,93,94,95,96,97,98]. Common adverse events include mild injection site reactions and conjunctivitis, which can occur in 5–30% of patients [99,100,101]. Transient eosinophilia has also been observed but is typically asymptomatic and resolves without intervention [102]. These agents do not induce broad immunosuppression, significantly reducing the risk of opportunistic infections; however, mild upper respiratory tract infections, herpes simplex virus reactivation, and rare cases of eczema herpeticum have been reported [103,104,105,106,107]. Still, they are considered as a safe alternative in populations at risk for infections.

A key question is whether therapies that target only IL-13, such as tralokinumab and lebrikizumab, possess the same disease-modifying potential as dupilumab [74,108]. Dupilumab’s dual mode of action allows it to inhibit both IL-13, the primary driver of skin inflammation, and IL-4, which plays a central role in IgE sensitization. Blocking IL-13 in the skin yields multiple benefits, including a reduction in inflammation and pruritus, improved skin barrier function, and decreased permeability to allergens, thereby mitigating the IgE sensitization processes. However, dupilumab simultaneously inhibits IL-4 produced by ILC2s. This dual targeting reduces not only local skin inflammation but also the systemic effects of cytokines and chemokines that circulate in the bloodstream, preventing the generation of allergen-specific IgE and the development of other allergic comorbidities [72].

#### 3.1.2. The OX40/OX40L Pathway

**Figure 6:**
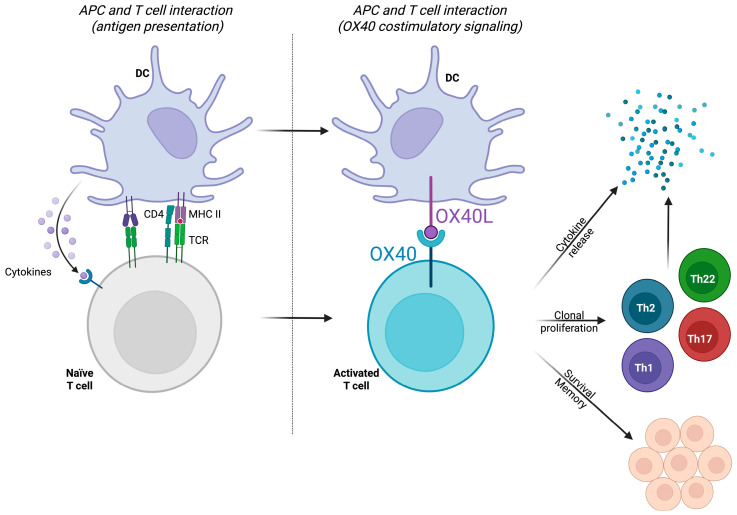
OX40/OX40L Axis. These molecules belong to the tumor necrosis factor (TNF) α receptor superfamily. OX40 (CD134) is a costimulatory molecule expressed on activated T cells, including both effector T cells and Tregs. OX40 displays a dual function in health and disease. Under physiological conditions, it modulates Tregs to maintain immune balance and prevent autoimmunity. In contrast, in AD, OX40 activation impairs Treg function, allowing inflammatory processes to persist unchecked. Its expression begins 24–72 h after T cells encounter an antigen, where it becomes essential for sustaining T cell activation and survival. The ligand for OX40, OX40L (CD134L/CD252), is expressed in various antigen-presenting cells (APCs) such as DCs, Langerhans cells, and macrophages. Additionally, activated B lymphocytes and T lymphocytes can also express OX40L, further amplifying immune responses through a positive feedback loop. Although OX40L expression is not constitutive, it can be induced via multiple pathways, including CD40 and TLR signaling, as well as cytokines like IL-18 and TSLP, which are released in response to barrier dysfunction or tissue damage. This pathway also facilitates the generation of memory T cells, ensuring rapid immune responses upon re-exposure to allergens [111]. From left to right: antigen presentation acts as the first signal in T cell activation. The interaction occurs between APCs—like DCs—and T cells (left panel). Once the T cell is activated, it begins to express OX40 (CD134), a costimulatory molecule. The APC expresses the ligand for OX40, known as OX40L (CD134L). The binding of OX40 to OX40L provides a second signal, which is critical for sustained T cell activation, clonal proliferation, and differentiation into effector subtypes (right panel).

The OX40-OX40L interaction has gained attention in recent years [109,110,111,112]. The OX40-OX40L axis not only promotes the expansion of Th2 cells but also ensures their prolonged survival and function in the affected tissue, contributing to the chronic nature of AD (Figure 6). Consequently, the OX40/OX40L pathway amplifies immune responses and perpetuates inflammation through sustaining T-cell activation and memory. Targeting this pathway modulates Th2 cell proliferation and function, alleviating inflammation and clinical symptoms [110,111,112]. Many therapies targeting this pathway are currently being explored. Telazorlimab (GBR-830; ISB-830) is an anti-OX40, currently in phase II of development. It has demonstrated efficacy in reducing symptoms of moderate-to-severe AD, significantly improving indices such as EASI and lowering inflammatory markers [113,114]. Rocatinlimab (AMG-451; KHK-4083), also an anti-OX40, is in phase III and has shown sustained reductions in disease severity after 56 weeks of treatment, with benefits persisting even after treatment discontinuation [115,116,117]. Also in phase III is amlitelimab (KY-1005; SAR-445229), an anti-OX40L. It is another promising option for modulating immune responses with long-lasting effects, decreases in serum levels of IL-13, and a favorable safety profile [115,118,119].

#### 3.1.3. Other Extracellular Immune Mediators and Targets

Tezepelumab (AMG 157; MEDI-9929) targets TSLP, a master regulator of type 2 immune responses, potentially affecting a broader range of inflammatory pathways. Its efficacy in AD, supported by a phase 2a clinical trial, appears less robust compared to dupilumab, tralokinumab, and lebrikizumab, and has allegedly been discontinued for AD. Bosakitug (BSI-045B; TQC-2731), has a similar mechanism of action and is still being studied. PF-07275315 is a mixed anti-IL-4/IL-13/TSLP molecule under development by Pfizer. After a phase I trial in volunteers in USA and Belgium, there has been an ongoing phase-II clinical trial in USA (NCT05995964) and an additional pharmacokinetics phase I trial since January 2025 (NCT06675188) for intravenous use.

Together with TSLP, IL-33 has also received interest as a target modulating activation of Th2 and ILCs. Itepekimab (REGN-3500; SAR-440340), etokimab (ANB-020), and tozorakimab (MEDI-3506) are IL-33 inhibitors which have been discontinued for AD but are still being studied for pulmonary diseases. Astegolimab (AMG-282; MSTT 1041 A; RG 6149; RO 7187807) is a fully human IgG monoclonal antibody that targets the ST2 receptor, thereby inhibiting IL-33 signaling. It did not demonstrate a significant clinical benefit over a placebo in the treatment of moderate to severe atopic dermatitis in its phase 2 trial [120]. PF-07264660 is a combined IL-4/IL-13/IL-33 inhibitor in an early phase of development in the pipeline of Pfizer.

CD1a is a lipid-presenting molecule highly expressed on Langerhans cells in the skin, playing a crucial role in presenting self and foreign lipids to T cells, thereby contributing to the pathogenesis of inflammatory skin diseases, including AD [121]. PF-07242813 was proposed for exploring this target as a treatment for AD but was discontinued in its early stages.

Sphingosine-1-phosphate receptor (S1PR) and CC Motif Chemokine Receptor (CCR) 4 have also been studied as potential targets. S1P is a bioactive lipid that regulates immune cell trafficking, including T-cell migration. S1P exerts its effects through five G-protein-coupled receptors (S1PR1-S1PR5). In the context of AD, S1PR modulation has shown promise in preclinical models [122]. CCR4 is a chemokine receptor highly expressed in Th2 and Th17 cells. The ligands for CCR4, CCL17, and CCL22 are upregulated in AD skin lesions and promote the recruitment of Th2 cells. Various modulators of these targets have been investigated, including etrasimod (PF-07915503; APD-334), vibozilimod (SCD-044), and zectivimod (LC-510255; LR-19019), BMS-986166. They are in different stages of development, without recent updates. Zelnecirnon (FLX-193, RPT-193), an anti CCR4, has been discontinued.

Rezpegaldesleukin (LY-3471851, NKTR-358) is a Treg-selective IL-2 receptor agonist designed to enhance Treg function and numbers, thereby modulating the immune response in inflammatory diseases like AD. In two randomized, double-blind, placebo-controlled phase Ib trials, rezpegaldesleukin recently demonstrated significant clinical improvements in patients with moderate-to-severe AD [123]. The treatment was well-tolerated, with a favorable safety profile and consistent pharmacokinetics. The clinical improvements were accompanied by sustained increases in CD25bright Tregs, indicating effective modulation of the immune response. A phase IIb clinical trial (REZOLVE-AD, NCT06136741) completed target enrollment as of January 2025 and released its efficacy and adverse event data in September. The 52-week maintenance data is expected in early 2026.

### 3.2. “JAK Inhibitors” Family

JAK inhibitors are immunomodulators that target intracellular signaling. Upadacitinib, abrocitinib, and baricitinib are JAK inhibitors approved by international regulatory bodies for the treatment of AD. As they block the JAK/STAT pathway, they are involved in broader cytokine signaling modulation. This is effective even for itch-dominant or refractory cases and provides faster symptom improvement [63,124]. Consequently, they could be considered for rapid relief of severe itching, chronic cases with mixed features and multiple immunologic axis involvement, cases compromising head-and-neck, or in cases with coexisting immune-mediated diseases (e.g., alopecia areata [125], dermatitis herpetiformis [126], or psoriasis [127]). They can also be considered as an option for patients with inadequate responses to monoclonal antibodies [63]. JAK inhibitors exhibit varying degrees of selectivity for different JAK isoforms, and some have higher affinity for specific JAK subtypes. The spectrum of affinities for drugs approved or under study in dermatology is represented in Table 1.

Apart from those already in the market, many molecules targeting this pathway are being studied. Beyond upadacitinib and abrocitinib, ivarmacitinib (SHR-0302) is another JAK1 selective that is currently in preregistration for its use in AD. Similarly, povorcitinib (INCB-54707) also targets JAK1 and is currently in phase III trials for prurigo nodularis and other immune mediated diseases, including hidradenitis suppurativa and vitiligo, and is in phase II for asthma and chronic urticaria. Lepzacitinib (ATI-1777) and ifidancitinib (A 301; ATI 50002) are other JAK1/JAK3 molecules being investigated, although the latter seems to have been discontinued from the Rigel Pharmaceuticals and Aclaris Therapeutics pipelines. Gecaxitinib (DB17545) is a pan-JAK in development for many indications, including AD. Cerdulatinib (ALXN 2075; DMVT 502) which, apart from being a pan-JAK, is a spleen tyrosine kinase (Syk) inhibitor, has been discontinued from the dermatologic pipeline but is still being studied for hematologic malignancies, whereas gusacitinib (ASN002), with a similar mechanism of action, is in late phases of clinical trials for eczematous dermatoses. In a study by Pavel et al., gusacitinib significantly suppressed key inflammatory pathways implicated in AD pathogenesis, including the Th2, Th17/Th22, and Th1 axes, and improved epidermal barrier markers [128]. These molecular changes correlated with clinical improvements in AD severity and pruritus. Another study demonstrated its superiority to a placebo in achieving EASI50 and EASI 75 responses, with dose-dependent efficacy and a favorable safety profile [129].

JAK inhibitors have demonstrated significant efficacy in the treatment of moderate-to-severe AD, offering rapid and robust symptom control by targeting key cytokine pathways involved in the disease’s pathogenesis, including IL-4, IL-13, IL-31, IFN-γ, and IL-22 [130,131,132]. Clinical trials have shown that JAK inhibitors achieve high rates of EASI-75 responses, with up to 70% of patients reaching this milestone within 12–16 weeks of treatment, significantly outperforming placebos and often demonstrating comparable or superior efficacy to biologics [133,134]. Additionally, JAK inhibitors rapidly alleviate pruritus, with improvements in itch severity reported as early as the first week of treatment, contributing to enhanced quality of life for patients. These outcomes underscore the potency of JAK inhibitors in addressing both the inflammatory and symptomatic burden of AD, establishing them as a valuable option in the therapeutic arsenal for moderate-to-severe disease.

Several systematic reviews, meta-analyses, and network meta-analyses have evaluated the safety of JAK inhibitors in AD by analyzing data from randomized controlled trials (RCTs). Compared with placebos, there is a higher risk of headaches, acne (particularly with abrocitinib and upadacitinib), nausea (notably with abrocitinib) and dose-dependent elevations of creatine phosphokinase (CPK) [135,136]. No significant increase in risk was found regarding serious infections or opportunistic infections, but the incidence of herpes zoster was significantly higher, as was, in some studies, that of herpes simplex [135,137]. The same allegedly applies for non-melanoma skin cancer (NMSC), other malignancies, and for major adverse cardiovascular events, but most studies that included these had short follow-up periods (12–16 weeks), limiting their understanding of these long-term risks [135,136]. An umbrella review has consolidated the findings from 16 meta-analyses regarding efficacy and safety. High dose abrocitinib and upadacitinib were considered especially effective, though they carried a higher risk of side effects [131]. Common ones included acne, nasopharyngitis, headaches, and gastrointestinal disturbances (nausea once again being prominent with abrocitinib) [131]. Again, the short duration of most trials limits the understanding of long-term safety [131]. Additionally, RWD on the safety of these treatments for adolescents and children still remain sparse [131,135].

### 3.3. Comparisons Between Families: What We Know So Far

RWD is only beginning to emerge in AD compared to other immune-mediated diseases [138,139], as it has a shorter trajectory in terms of advanced therapeutic options [140,141,142,143]. Nevertheless, the field is rapidly evolving, and the increasing availability of approved therapies together with a constantly expanding pipeline underscores the importance of robust real-world comparisons to ensure the optimal use of resources while delivering personalized care [8,144]. This is particularly significant in AD, as it has a high placebo response rate, as seen in many of the already-available clinical trials [145]. To date, comprehensive comparative analyses of the use of biologics and JAK inhibitors in real-world settings are still limited or are preliminary in nature [101,146]. Clinical trial populations are highly selected and relatively homogeneous, which may permit post hoc indirect comparisons of treatments under controlled conditions [124,147]. However, this uniformity does not capture the heterogeneity and complexity of patients encountered in everyday clinical practice. As a result, meaningful comparisons—whether between real-world data (RWD) and prior randomized controlled trials (RCTs), or between different treatments in real-world settings—largely depend on how well the characteristics of patients treated after drug approval are defined and reported [124,148,149,150]. This is essential to assess how these treatments are being implemented in real-world practice and whether current strategies are aligned with the aim of optimizing clinical outcomes as well as resource utilization. Increasing reports suggest a disconnection between theoretical assumptions—based on clinical trial data—about ideal patient profiles for each group of medications, particularly regarding atopic comorbidities and cardiovascular risk factors [63,130,151,152,153,154] and how advanced therapies are actually being prescribed in real-world settings [124,138,149]. This highlights the need to better understand the real-life drivers of treatment selection beyond controlled environments. Collaborative efforts to accurately characterize patient populations across settings are essential to improve the extrapolation of real-world data and enable meaningful comparisons of drug efficacy and safety.

No head-to-head direct comparisons have been published among advanced systemic therapies. However, several reviews and meta-analyses provide indirect comparative efficacy and safety data. A systematic literature review and network meta-analysis published in 2021 found that 30 mg of upadacitinib once daily had the highest numerical efficacy, followed by 200 mg abrocitinib once daily, and 300 mg dupilumab every 2 weeks [146]. A network meta-analysis also pointed to the higher efficacy of 200 mg abrocitinib and also that of upadacitinib at 15 mg daily over dupilumab and lebrikizumab, both of which showed similar efficacy in the absence of topical corticosteroids (TCS) or calcineurin inhibitors [155]. However, more recently, in 2023, a systematic review and meta-analysis compared data from 37 RCT, involving 18,172 participants and highlighting dupilumab and upadacitinib at 30mg due to their efficacy [139]. Lastly, this past year, another network meta-analysis compared binary efficacy outcomes and found that 30 mg upadacitinib daily and 200 mg abrocitinib daily were associated with higher odds of achieving EASI 50 compared to dupilumab, whereas baricitinib and tralokinumab were associated with lower odds of achieving EASI 50 compared to dupilumab [156].

Most treatments available have exhibited favorable safety profiles with few AEs and minimal to non-existent serious ones. In certain pools of data, baricitinib showed a slightly increased risk of severe AEs at higher doses compared to other treatments [139]. Some authors have further analyzed and compared the safety of these groups of treatments based only on real-world evidence [138]. In the short- and medium-term, oral JAK inhibitors were not associated with increased risks of mortality, malignancies, major adverse cardiovascular events, venous thromboembolism, renal events, or serious gastrointestinal events [138]. However, patients receiving JAK inhibitors showed significantly higher risks of acne (HR 2.09), cytopenia (anemia HR 1.83, neutropenia HR 4.0, thrombocytopenia HR 1.76), hyperlipidemia (HR 1.45), skin and subcutaneous tissue infection (HR 1.35), herpes simplex reactivation (HR 1.64), and herpes zoster (HR 2.51) [138]. Indeed, biologics and JAK inhibitors differ in their specific infection profiles, influenced by their mechanisms of action. JAK inhibitors, particularly at high doses and non-selective ones, carry a higher risk of herpes simplex, herpes zoster, and hepatitis B reactivation compared to biologics [136,157]. Considering malignancies, a literature review analyzed various studies and trials conducted from 2010 to 2024, focusing on concretely on skin and non-skin cancer risks [158]. Both biologic drugs and JAK inhibitors are generally safe regarding cancer risk, with incidences comparable to the general population in dermatological patients. The risk, however, seems to be higher in patients treated for rheumatoid arthritis or other systemic inflammatory diseases [158].

Based on the above, consideration of patient age, disease systemic inflammatory burden, comorbidities and individual risk of infection is critical for dermatologists in assessing individual threats as we still lack enough long-term, prospective, head-to-head, real-world studies. Meaningful and accurate comparisons among treatments’ outcomes built upon unselected cohorts from the clinical setting with proven similarities in their baseline characteristics will be key for selecting future treatments. JAK inhibitors, due to their short half-life and rapid onset of action, can be used flexibly, including in intermittent regimens for conditions like seasonal dermatitis, where short-term control is sufficient. In contrast, biologics require continuous and uninterrupted treatment to maintain their efficacy due to their longer half-life and the gradual buildup of their therapeutic effects. Monitoring requirements also vary; biologics generally require no routine laboratory monitoring, making them a convenient choice for long-term management, whereas JAK inhibitors necessitate stricter surveillance, including baseline and periodic assessments of their lipid profiles, liver enzymes, blood counts, and thrombosis risk, due to their broader immunomodulatory effects. With biologics, primarily targeting type 2 inflammation, there is a risk of Th1 skewing over time, potentially contributing to unexpected immune dysregulation, though this remains a topic of ongoing investigation. The route of administration differs, with biologics delivered via subcutaneous injections, requiring patient education or clinic visits, while JAK inhibitors are oral medications, offering ease of use and adherence, particularly for patients who prefer non-invasive therapies.

Another relevant difference among drug families and individual drugs is the time needed to evaluate treatment response, which can vary significantly. Therapeutic response is typically assessed clinically at week 16 for biologics and at week 8–12–16 for baricitinib, upadacitinib, and abrocitinib [146,159,160,161,162,163,164,165]. Despite that, earlier signs of improvement in pruritus and inflammation can be visible in the first weeks with monoclonal antibodies and from the very first days with JAK inhibitors. Another topic yet to be fully elucidated is the timing of disease relapse after cessation of treatment. In a recent study on a cohort of 83 adolescent Chinese patients receiving upadacitinib or dupilumab, following the discontinuation of therapy, Kaplan–Meier analysis revealed a median time to relapse of 270 days in the dupilumab group compared to 18 days in the upadacitinib group [166].

All these considerations should be taken into account, plus additional individual factors such as disease phenotype [28] or, for example, those patients simultaneously undergoing allergen immunotherapy. Allergen immunotherapy promotes tolerance primarily through the induction of allergen-specific regulatory T cells and immune deviation toward non-pathogenic responses—mechanisms that may be compromised by broad immunosuppressive agents [167,168].

Additionally, novel alternatives might help to fill the remaining gaps. Nemolizumab is a monoclonal antibody targeting IL-31R, which has managed to deliver a substantial improvement in pruritus in RCTs and exhibits an acceptable safety profile, with no significant differences in most adverse event rates compared to a placebo [169,170,171,172,173]. Although it has significantly improved EASI, SCORAD, and BSA scores in patients treated in RCTs, it might also be used as a coadjutant to other therapies for managing incoercible pruritus in conventional AD patients [71,170,174,175,176]. Vixarelimab (KPL-716; RG-6536; RO-7622888) is a monoclonal antibody that inhibits OSMRβ, blocking IL-31 signaling. It is currently being explored for prurigo nodularis and pruritus, among other indications. Ruxolitinib cream, among other topical JAK inhibitors, for example, is already recommended for mild-to-severe AD as an alternative with favorable safety profile and low risk of discontinuation due to adverse effects [130,177,178]. The possibility of combinations between systemic therapies with topical JAK inhibitors, if economically feasible, could be another promising approach for managing AD [139]. Given the range of therapeutic options available following inadequate response to prior advanced systemic treatments, some authors have even proposed mathematical models to identify optimal combinations of drug targets [179].

### 3.4. Alternative Approaches

#### 3.4.1. Other Interleukins

IL-1α is a cytokine involved in inflammatory processes. Bermekimab (CA-18C3; MAB-p1) was a human IgG1 monoclonal antibody specific for human IL-1α. It showed potential in early-phase trials for atopic dermatitis, but larger controlled studies did not confirm its efficacy, leading to the early termination of some trials [180].

GSK1070806 is an IL-18 inhibitor that is under development and is reportedly at phase II for AD. IL-18 is an interesting cytokine that acts as an alarmin and belongs to the IL-1 family. So far, patients with moderate-to-severe AD who received GSK1070806 experienced positive improvements across all PROs versus a placebo after 12 weeks [181]. A long-term study is currently ongoing (NCT06447506). KT-474 (SAR 444656) is an IRAK4 degrader. It targets the TLR/IL-1R, MyD88 and IRAK4 signaling axis. Therefore, its mechanism is more specific to innate immune signaling and downstream activation, and it was discontinued for AD.

IL-22, involved in keratinocyte hyperproliferation and epidermal barrier disruption, is targeted by monoclonal antibodies such as fezakinumab (PF-5212367; ILV-094). These therapies were particularly promising for patients with chronic, lichenified lesions. However, while fezakinumab showed significant clinical improvements in early-phase trials back in 2018, its current developmental stage seems to have been stopped in phase IIa. Similarly, temtokibart (LEO 138559; LP-0145), an IL-22 receptor antagonist directed against the IL-22Rα1 subunit, is under investigation in phase IIb. Efficacy and safety data from its phase IIa trial was presented at the 2023 AAD Annual Meeting, and pharmacodynamics data from a phase IIb trial has recently been released by LEO Pharma.

#### 3.4.2. Additional Kinase Inhibitors

The potential of Bruton’s Tyrosine Kinase (BTK) inhibitors in allergic disorders has recently begun to be explored. The broad inhibition of the high-affinity IgE receptor (FcεRI) pathway—expressed on the surface of mast cells, basophils, and, to a lesser extent, eosinophils, DC, and Langerhans cells—by BTK inhibitors suggests some potential [182]. Branebrutinib (BMS-986195) is a covalent, irreversible inhibitor of BTK that has shown promise in early clinical trials for various immune-mediated conditions. In a Phase I study, branebrutinib demonstrated a favorable safety profile in healthy participants, with adverse events being mostly mild to moderate. The drug was rapidly absorbed, achieving maximum plasma concentration within 1 h, and exhibited a short half-life of 1.2–1.7 h. Importantly, branebrutinib achieved 100% BTK occupancy after a single 10 mg dose, with sustained pharmacodynamic effects despite rapid plasma clearance.

BEN-2293, a tropomyosin receptor kinase (TRK) antagonist, has been under investigation in recent years for the treatment of AD but, as of the latest available data, there are no published clinical trials, and its development is likely suspended.

#### 3.4.3. Eosinophils and IgE

Emerging treatments for AD that target eosinophils include monoclonal antibodies that either directly bind to IL-5 (mepolizumab and reslizumab) or block its receptor (benralizumab). Their action—inhibiting eosinophils’ growth, differentiation, recruitment, activation, and survival—reduces cell counts and supposedly mitigates their role in the inflammatory process of atopic dermatitis [183]. Benralizumab targets the IL-5 receptor alpha (IL-5Rα) on eosinophils and basophils, inducing antibody-dependent cell-mediated cytotoxicity (ADCC), leading to the rapid depletion of eosinophils. However, despite eosinophil reductions, the clinical improvements achievable by these drugs seem to be limited [184].

Omalizumab (RG-3648), a monoclonal antibody that binds to free IgE, has shown efficacy in treating recalcitrant AD [185]. The ADAPT trial demonstrated that omalizumab significantly reduced AD severity and improved quality of life in pediatric patients, with a corticosteroid-sparing effect. As of today, no registration or preregistration for this indication has been carried out. Anti-CemX (FB 825) targets membrane-bound IgE (mIgE) on B cells, lysing mIgE-expressing B lymphoblasts and preventing the generation of IgE-producing plasma cells. This mechanism allows it to modulate the IgE pathway regardless of serum IgE levels. A study published in 2016 suggested that anti-CemX could offer clinical benefits in IgE-mediated diseases, including AD, by providing a different clinical utility compared to traditional anti-IgE therapies [186]. However, since then, no new information supporting this mechanism has become available. Further clinical data would be needed to establish the role of this novel approach.

## 4. Conclusions

By targeting the precise molecular and cellular pathways involved in AD, emerging therapies—such as biologics, JAK inhibitors, and modulators of key immune and neural pathways—can effectively alleviate symptoms and in some cases, potentially, modify the course of the disease. These advanced approaches hold promise for breaking the cycle of inflammation, preventing disease progression, and reducing the burden of AD and its associated comorbidities, and ultimately improving the quality of life for those affected by this debilitating condition.

## Figures and Tables

**Table 1:** Known pharmacological selectivity profiles of JAK inhibitors marketed and under investigation in dermatology across JAK isoforms, including JAK1, JAK2, JAK3, and TYK2. Checkmarks (✓) indicate the isoforms for which each inhibitor exhibits significant affinity or activity. This categorization highlights the differential mechanisms of action and therapeutic potential of each JAK inhibitor in modulating inflammatory and immune responses. PO: *per os* (oral administration).

	JAK1	JAK2	JAK3	TYK2	Administration Route in Dermatology
Upadacitinib	✓				PO
Abrocitinib	✓				PO
Ivarmacitinib	✓				PO
Povorcitinib	✓				PO
Baricitinib	✓	✓			PO
Ruxolitinib	✓	✓			Topical
Tofacitinib	✓		✓		PO
Lepzacitinib	✓		✓		Topical
Ifidancitinib	✓		✓		Topical
Ritlecitinib			✓		PO
Deucravacitinib				✓	PO
Brepocitinib	✓			✓	PO/Topical
Zasocitinib				✓	PO
Delgocitinib	✓	✓	✓	✓	Topical
Gecaxitinib	✓	✓	✓	✓	PO
Cerdulatinib	✓	✓	✓	✓	Topical
Gusacitinib	✓	✓	✓	✓	PO

## Data Availability

No new data were created or analyzed in this study. Data sharing is not applicable to this article.
